# Effect of Light Emission Through an Optical Fiber Device on the Bond Strength of a Hollow Experimental Intraradicular Post

**DOI:** 10.3290/j.jad.b3240659

**Published:** 2022-08-18

**Authors:** Cristiane Mayumi Inagati, Manassés Tercio Vieira Grangeiro, Natália Rivoli Rossi, João Paulo Mendes Tribst, Leonardo Jiro Nomura Nakano, Paula Carolina Komori de Carvalho, Tarcisio José de Arruda Paes Junior

**Affiliations:** a PhD Student, Department of Dental Materials and Prosthodontics, São Paulo State University (UNESP), Institute of Science and Technology, São José dos Campos, SP, Brazil. Idea, hypothesis, experimental design, performed the experiments in partial fulfillment of requeriments for a degree, performed statistical evaluation, wrote and proofread the manuscript, contributed substantially to discussion.; b PhD Student, Department of Dental Materials and Prosthodontics, São Paulo State University (UNESP), Institute of Science and Technology, São José dos Campos, SP, Brazil. Helped perform push-out testing, performed statistical evaluation, proofread the manuscript.; c PhD Student, Department of Dental Materials and Prosthodontics, São Paulo State University (UNESP), Institute of Science and Tecnology, São José dos Campos, SP, Brazil. Helped performed a conversion degree analysis experiment, translate to English, proofread the manuscript.; d Professor, Department of Dental Materials, University of Amsterdam and Vrije Universiteit Amsterdam, Academic Centre for Dentistry Amsterdam (ACTA), Amsterdam, The Netherlands. Performed finite element analysis, proofread the manuscript.; e PhD Student, Department of Dental Materials and Prosthodontics, São Paulo State University (UNESP), Institute of Science and Technology, Brazil. Helped conduct the cementation experiment, proofread the manuscript.; f Professor, Department of Dental Materials and Prosthodontics, São Paulo State University (UNESP), Institute of Science and Technology, Brazil. Idea, experimental design, supervision, proofread the manuscript, contributed substantially to discussion.; g Associate Professor, Department of Dental Materials and Prosthodontics, São Paulo State University (UNESP), Institute of Science and Technology, São José dos Campos, SP, Brazil. Idea, hypothesis, experimental design, supervision, proofread the manuscript, contributed substantially to discussion, project administration.

**Keywords:** dental posts, finite element analysis, light-curing of dental adhesives, luting, optical fiber.

## Abstract

**Purpose:**

To evaluate the effect of irradiation with an optical-fiber device on the bond strength of hollow and partially opaque intraradicular posts.

**Materials and Methods:**

An optical-fiber accessory tip was attached to a light-curing unit to emit light through the central hollow of an experimental fiberglass post. The samples were divided into 4 groups (n = 80) according to the protocol (Variolink N [light cured] or Multilink N [dual-curing luting material]) and the light-curing mode (performed conventionally or with the optical fiber): GF: light-curing luting material; GFF: light-curing luting material and optical fiber; GD: dual-curing luting material; GDF: dual-curing luting material and optical fiber. The samples were tested immediately or after aging. Push-out bond strength, failure mode, degree of conversion (DC, assessed at the peak of 1750 cm^-1^), and stress distribution by finite element analysis were performed. Quantitative data were analyzed using 3-way ANOVA (luting material x light curing x depth) and 2-way ANOVA (aging x luting material), followed by Tukey’s test.

**Results:**

Bond strength was significantly affected by the luting material protocol (p < 0.001), depth (p = 0.010), and light curing mode (p = 0.031). The GFF group revealed higher bond strength in the middle and apical portions. The most frequent failure modes were adhesive in the apical portion for the GFF and GDF groups. The DC was higher for GF and GFF groups.

**Conclusion:**

Using the optical-fiber device led to superior bond strength results when a dual-curing luting material was used.

Light-emitting diodes (LEDs) emerged in the 1990s as light sources useful for promoting the bonding between the restorative material and dentin substrate.^18^ Compared to other light sources, their major advantages in restorative treatment are narrow wavelength, lower heat emission, lower maintenance cost, and ability to increase restoration longevity.^11,14^ However, for restoration success, it is necessary for the light to activate the restorative material, in an adequate time and suitable wavelength range.^4,15^ When insufficient light reaches the resin, it can lead to biomechanical, physical, and biological problems as well as poor conversion of the monomers to polymers, which alters the final quality and longevity of the restoration.^25,27^

A major concern of dentists lies in the fact that the greater the restoration depth, the weaker the photoactivation.^15^ Endodontically treated teeth often need intraradicular posts to support the crown structures due to extensive loss of dental tissue.^7,17^ Several post options exist, but the most popular are prefabricated fiberglass posts, as they present an elastic modulus which is closer to that of dentin tissue, chemical characteristics compatible with the adhesive, and favorable esthetics.^7,17^ In addition, efficaceous luting material and adequate light curing are necessary to achieve satisfactory bond strengths between the dentin substrate and the post.^7,8,17^

There are basically three types of resin cements based on their activation mode: chemically curing, light curing, and dual curing (cured by a combination of the two methods). Chemically-activated luting materials are usually indicated in intraradicular post restorations, as there is no need for light curing, which reduces the problem of lower polymerization in the apical region; however, this luting material type is the most difficult to handle.^5-7,17,19^ While light-curing cements provide total control of their working time and moisture, their polymerization at greater depths is limited.^5,29^ Dual-curing cements are generally the most versatile cements, can be used in different clinical situations, and provide a cost-effective option, as they combine the characteristics of a chemically luting with a photoactivated luting material.^29^ Although dual-curing luting material is sometimes the primary choice of many professionals, they have acid-base components which can interfere in the bond quality when in contact with dental tissue.^3^

As a result of the various concerns about luting the intraradicular post, it is necessary to develop and improve post designs to allow complete photoactivation inside the root canal. Using materials with a certain translucency, the post can present higher bond strength, especially at the cervical level.^17^ However, in the apical portion of the root, the bond strength to fiberglass posts is still suboptimal.

Based on the need to increase the bond quality in the apical portion, the objective of this study was to evaluate an alternative light-curing mode with the aid of an optical-fiber accessory tip for light emission in a hollow prefabricated post. The tested hypotheses were: 1) the bond strength of the experimental groups would present results similar to that of the control group; 2) aging simulation would decrease the bond strength regardless of the luting protocol; and 3) the light-curing and dual-curing cements would show similar mechanical behavior.

## MATERIALS AND METHODS

### Accessory Device for Light Emission

**Fig 1:**
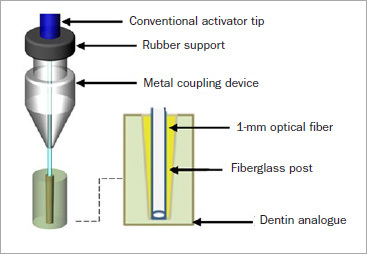
Schematic illustration of light emission assisted by optical fiber coupled to fiberglass post.

A device for light transmission was made of optical fiber with the purpose of acting together with the experimental glass-fiber post. The device was customized to be inserted inside the post and in conjuction with conventional light curing would increase polymerization in the apical portion (Fig 1). The optical-fiber accessory tip was developed in partnership with the Photonita Optical Metrologia company (Santa Catarina, Brazil), and a patent request was submitted and approved by the Innovation Agency at the National Institute of Industrial Property (INPI).

**Fig 2:**
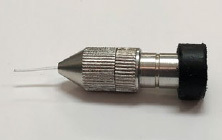
Accessory device in which the optical fiber is inserted and coupled to the light-curing unit.

The intensity of the light source was checked by measuring the irradiance of the optical-fiber device in comparison with the light-curing unit’s tip (Bluephase, Ivoclar Vivadent; Schaan, Liechtenstein), in order to verify the energy loss of the optical fiber. For its calculation, the NOVA light potentiometer (Optronics solutions; Jerusalem, Israel) was used. Figure 2 shows the optical fiber device that was used in the present study.

### Determination of Post-Hollow Diameter

In addition to effects of the light device, it was necessary to know if the central hollow in the fiberglass post would decrease its strength significantly. To this end, a 3D finite element analysis was performed to define the hollow’s diameter and evaluate the mechanical response compared to a solid, conventional post, to verify the feasibility and possibility of hollow-post manufacture that would provide adequate stress resistance during loading. Initially, a 3D model of the intraradicular post was designed with pre-set dimensions, based on an STL (stereolithography) file obtained after the digital scan of a commercial post using the Ceramill Map 300 Scanner (Amann Girrbach; Koblach, Austria). The generated STL file was exported to the CAD (Computer Aided Design) software to be edited according to the luting material and the use of optical fiber (Rhinoceros 5.0, McNeel North America; Seattle, WA, USA).

In the design software, the post was converted to a volumetric solid; polygons and surfaces were created based on the intersection of different lines in the network. The surfaces were then joined into solids to generate the volume of the post’s final dimensions.^28^ The prototype design had an internal canal of uniform diameter of 1.1 mm and a length of 10 mm, while the post wall thickness was 0.7 mm in the cervical region and 0.2 mm in the apical region.

The numerical simulation was applied to design the hollow’s diameter without increasing the stress concentration magnitude in comparison with a conventional solid post. Therefore, the central hollow was modelled with 4 different diameters (0.1 mm, 0.5 mm, 0.7 mm, and 1.0 mm diameters).

Then each post model was entered in a previous 3D model^2^ composed of periodontal ligament and cortical bone with a thickness of 0.3 and 0.5 mm (the medullary bone is represented as space surrounded by cortical bone) respectively, gutta percha with length of 4 mm, an all-ceramic crown of lithium disilicate (e.max CAD, Ivoclar Vivadent; Schaan, Liechtenstein), and dentin. The crown as well as the post and core were cemented with a 0.1-mm-thick cement layer.

These geometries were exported to the Computer Aided Engineering (CAE) software (ANSYS 16.0, Engineering Simulation and Scientific Software; Florianópolis, Brazil), where the convergence test was applied to subdivide the complex geometries into elements and nodes. The materials were considered isotropic, homogeneous, and linearly elastic with perfectly bonded contacts. An oblique (45-degree) load of 100 N was applied to the central region of the palatal face, with the system fixated at the cortical bone base.^2^ In this study, the stress distribution was analyzed by the Maximum Principal Stress criterion (tensile stress) and the results at the external surface of the post were considered to evaluate the effect of the hollow’s size.^28^

**Fig 3:**
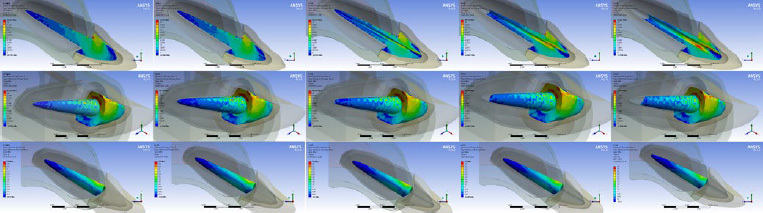
Stress distribution by finite element analysis as a preliminary investigation for determining the hollow’s diameter.

**Fig 4:**
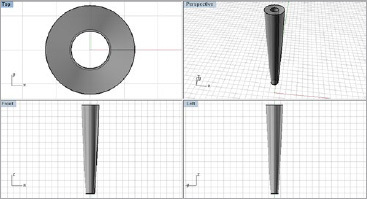
CAD drawing of the intraradicular post prototype that was subsequently manufactured.

After analysis, the highest stress concentration was observed in the core region of the conventional solid post (Fig 3); however, the stress concentration was similar in the root portion regardless of the diameter variation. In this sense, a 1.0-mm hollow will not increase the stress concentration in the external walls of the post and was selected as a parameter to manufacture the in-vitro samples (Fig 4).

### Post Manufacturing

**Fig 5:**
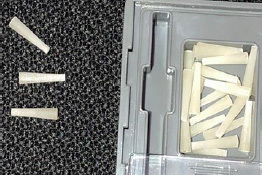
Hollow fiberglass posts manufactured according to the required dimensions.

Based on the CAD model, the fiberglass posts were manufactured in partnership with the Angelus company (Londrina, Paraná, Brazil). The post length was 10 mm (Fig 5) and fabricated with smaller dimensions than usual due to technical limitations reported by the company during manufacturing. The description of this product had a patent request and approval by the National Institute of Industrial Property (INPI). The mechanical properties of the prototypes were based on the characteristics of well-established brands of prefabricated fiberglass posts and the central hollow, which was designed using stress data from the tooth loaded by the fiberglass post.

### Specimen Preparation

For the in-vitro test, dentin analogues made of G10 resin (NEMA [National Electrical Manufacturers]; Arlington, VA, USA) were used, which is a widely used polymeric material due to its similarity to dentin in terms of elastic modulus, as previously demonstrated.^12,20^ Bond strength was tested between the surface of the G10 resin and the experimental post using different resin cements. For that, a circular piece of G10 was cut to form 80 specimens 10 mm wide and 14 mm high. A high-speed motor was fixed and positioned in the vertical dimension to drill a central space in the resin specimen using a diamond tip (Number 4138, KG Sorensen; Cotia, São Paulo, Brazil). The following perforation dimensions were obtained: diameter of 2 mm in the cervical portion, 1.6 mm in the apical portion and 10 mm in length for subsequent luting of the intraradicular post. The posts were cemented until reaching the central hollow’s maximum length of 10 mm, following a previous protocol which reported that there should be no gaps during luting to obtain greater mechanical strength.^7^ The samples were then washed in an ultrasonic bath for 15 min in distilled water to remove debris from the artificial root canal.

### Post Luting

The protocol established by the luting material manufacturer was followed for cementing the specimens. The intraradicular posts were cleaned with alcohol, and Monobond N silane (Ivoclar Vivadent) was applied on its external portion, then the product was allowed to evaporate for 1 min.

The G10 resin surface was treated with 37% phosphoric acid (Villevie; Blumenau, Santa Catarina, Brazil) for 30 s and then washed with water. The root canal was dried with an air jet, and an adhesive layer (Adper Single Bond 2 dentin adhesive, 3M Oral Care; St Paul, MN, USA) was applied with the aid of a microbrush, followed by an air jet and light curing (Bluephase, Ivoclar Vivadent) for 20 s (power: 1200 mW/cm^2^).

**Fig 6:**
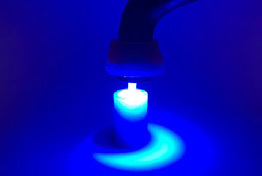
Light curing of a representative specimen directly onto the intraradicular post.

**Fig 7:**
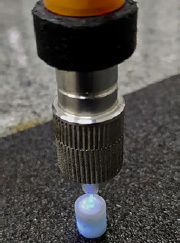
Light curing of a representative specimen with the optical fiber accessory tip through the hollow of the intraradicular post.

The fiberglass posts from each of the 4 groups received different types of treatment: dual-curing luting material (Multilink N, Ivoclar Vivadent) was used in the GD and GDF groups due to the option of additional light curing (Fig 6). Light-curing luting material (Variolink Ivoclar Vivadent) was used in the GF and GFF groups. For both cements, the manufacturers’ recommendations were followed. In the GFF and GDF groups, the luting material was applied on the post walls and was introduced into the artificial canal, together with the optical fiber. Then, light was emitted for 30 s or 20 s in the GFF or GDF groups, respectively; the emitting tip was retracted by about 2 mm and photoactivated for 30 s or 20 s and so it successively proceeded to the cervical portion of the root (Fig 7).

### Thermocycling (Aging Simulation)

**Fig 8:**
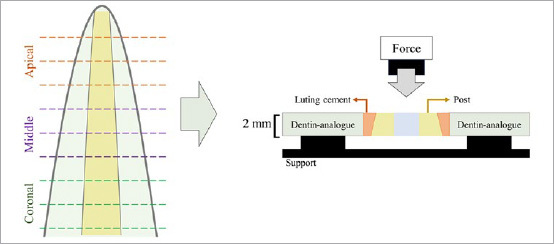
Schematic drawing of the distribution of root slices, with nine 2-mm-thick slices (3 per third) obtained from each root sample. Schematic diagram of the push-out test procedure on the tooth slices.

To artifically age the specimens, half (N = 40) were subjected to thermocycling at temperatures of 5°C and 55°C for 10,000 cycles in a thermal cycler (Biopdi; São Carlos, São Paulo, Brazil) to evaluate the adhesive interface degradation over time. After aging, the specimens were sectioned transversely into 2-mm segments using an Isomet 1000 metallographic cutter (Buehler; Lake Bluff, IL, USA) to be later submitted to the push-out bond strength test in 3 regions of the root (coronal, middle, and apical) (Fig 8).

### Push-out Bond Strength Test

For aged and non-aged specimens, the push-out tensile test was used to assess the bond strength between the luting material, post, and dentin analogue. The test was performed on a universal testing machine to determine maximum fracture load resistance (EMIC DL 1000; Joinville, Brazil), applying a load cell of 100 kgf at a speed of 1.0 mm/min. The bond strength was recorded in Newton (N) and converted to MPa. Bond strength in MPa was calculated as F/A, in which F = force (N), A = area (mm^2^), A = (B+b)h/2, in which: B = larger base (mm), b = smaller base (mm), h = height (mm).

### Failure Analysis

Failures modes were evaluated using a stereomicroscope (Carl Zeiss, Discovery V20; Oberkochen, Germany) and classified according to the literature:^22^ adhesive between G10 and luting material (AG10-L); adhesive between post and luting material (AP-L); mixed (M) when part of the luting material adhered to the substrate; and cohesive in G10 (CG10).

### Degree of Conversion (DC)

Silicone impressions were made in a 1-mm-thick circular shape, where 3 samples of Variolink and Multilink N luting material were prepared and light cured with or without the optical fiber according to the methodology described above. Then Fourier Transform Infrared Spectroscopy (FTIR) was performed using a Spectrum One spectrophotometer (Perkin Elmer; Waltham, MA, USA) and analyzed using the attenuated total reflectance (ATR) technique to perform 16 scans in the 2000-500 cm^-1^ range with a resolution of 4 cm^-1^.

### Statistical Analysis

The independent variables of this study were the luting material types (dual- or light-curing), light-curing mode (direct or with the optical fiber), and depth (coronal, middle, and apical). The dependent (response) variables were push-out tests, stereomicroscopic analysis, FTIR spectroscopy, and thermocycling were the dependent variables. A value of N = 10 was determined and the Minitab 17 statistical program was used for the analyses. The data set of the response variable was also initially evaluated regarding its distribution using the Kolmogorov-Smirnov test. Once the values showed normal distribution, a 3-way ANOVA was used for repeated measures (depth, fiber, and luting material), followed by Tukey’s test, with a significance level of 5%. The tests were analyzed separately regarding the specimens submitted to thermocycling using 2-way ANOVA for repeated measures (aging and luting material), followed by Tukey’s test.

## RESULTS

The push-out bond strength test showed statistically significant differences according to light-curing mode, type of luting material, and root depth. There was also an interaction between luting material type and depth factors (p < 0.05; 3-way ANOVA) (Table 1).

**Table 1:** Three-way ANOVA factors of the push-out test data

	df	SS	Ms	F	p-value
Light curing	1	337.4	337.41	4.75	**0.031**
Luting material	1	2885.5	2885.51	40.63	p < **0.001**
Depth	2	688.1	344.05	4.84	**0.010**
Light curing x luting material	1	11.3	11.32	0.16	0.690
Light curing x depth	2	340.1	170.06	2.39	0.096
Luting material x depth	2	652.5	326.27	4.59	**0.012**
Light curing x luting material x depth	2	364.1	182.04	2.56	0.082
Error	108	7669.8	71.02		
Total	119	12948.9			

p-values in bold indicate significantly different SBS (p < 0.05). df: degrees of freedom; SS: sum of squares; Ms: mean square.

Specimens light cured with the optical fiber device showed higher bond strengths than specimens cured conventionally (Table 2). Furthermore, bond strengths using the light-curing luting material surpassed those of the dual-curing luting material (Table 2). There was a difference between the light- and dual-curing luting materials in the interaction between luting material and depth, with the light-cured groups showing the highest bond strengths. The coronal region different significantly from the apical region in the GFF group (Table 3).

**Table 2:** Tukey’s test for push-out data in MPa on the use or not of the optical fiber device and luting material type

Fiber	Mean	N			Luting material	Mean	N		
Yes	65.1318 (10,53)	60	A		Light cured	68.3587 (8.37)	60	A	
No	61.7782 (10,14)	60		B	Dual	58.5513 (8.30)	60		B

*Different letters indicate statistically significant difference.

**Table 3:** Tukey’s test for push-out data (MPa) on the interaction between luting material and root thirds

Luting material	Depth	N	Mean
Light cured	Coronal	20	74.3165^a^ (8.77)
Light cured	Middle	20	67.8430^ab^ (8.37)
Light cured	Apical	20	62.9165^bc^ (9.70)
Dual curing	Coronal	20	59.0830^c^ (8.30)
Dual curing	Middle	20	57.5015^c^ (8.03)
Dual curing	Apical	20	56.0695^c^ (9.17)

Different superscript letters indicate statistically significant differences.

Fracture modes can be seen in Tables 4 and 5, which refer to immediate and aged groups, respectively. The adhesive failure mode between substrate and luting material was observed in groups light cured with the optical fiber, regardless of the luting material and aging. However, there was a predominance of adhesive failures in the GF group only in the coronal portion, but exhibited cohesive fractures in the G10 resin in the apical portion. In the GD group, mixed failures prevailed.

**Table 4:** Observed failure modes for immediate tests

Groups	Depth	AG10-L	AP-L	M	CG10
GF	Coronal	80%	0.0%	20%	0.0%
	Middle	10%	0.0%	70%	10%
	Apical	0.0%	0.0%	20%	80%
GFF	Coronal	80%	0.0%	20%	0.0%
	Middle	70%	0.0%	30%	0.0%
	Apical	60%	0.0%	20%	20%
GD	Coronal	90%	0.0%	10%	0.0%
	Middle	20%	0.0%	70%	10%
	Apical	20%	0.0%	70%	10%
GDF	Coronal	80%	0.0%	20%	0.0%
	Middle	60%	0.0%	40%	0.0%
	Apical	50%	0.0%	40%	10%

GF: light-curing luting material; GFF: light-curing luting material and optical fiber; GD: dual-curing luting material; GDF: dual-curing luting material and optical fiber; AG10-L: adhesive failure between G10 and luting material; AP-C: adhesive between between post and luting material; M: mixed; CG10: cohesive failure in G10.

**Table 5:** Observed failure modes after thermocycling

Groups	Depth	AG10-L	AP-L	M	CG10
GF	Coronal	100%	0.0%	0.0%	0.0%
	Middle	100%	0.0%	0.0%	10%
	Apical	10%	0.0%	20%	70%
GFF	Coronal	100%	0.0%	0.0%	0.0%
	Middle	100%	0.0%	0.0%	0.0%
	Apical	70%	0.0%	30%	0.0%
GD	Coronal	100%	0.0%	0.0%	0.0%
	Middle	90%	0.0%	10%	0.0%
	Apical	30%	0.0%	60%	10%
GDF	Coronal	90%	0.0%	10%	0.0%
	Middle	90%	0.0%	10%	0.0%
	Apical	70%	0.0%	30%	10%

GF: light-curing luting material; GFF: light-curing luting material and optical fiber; GD: dual-curing luting material; GDF: dual-curing luting material and optical fiber; AG10-L: adhesive failure between G10 and luting material; AP-L: adhesive failure between post and luting material; M: mixed; CG10: cohesive failure in G10.

**Fig 9:**
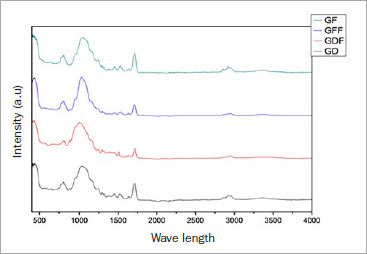
Fourier Transform Infrared Spectroscopy (FTIR) results of activation intensity.

The FTIR data are summarized in Fig 9, showing that the activation intensity was higher for the photoactivated luting material. The GD and GDF groups had the lowest degrees of conversion among the photoactivated groups, while the control group had the highest DC, but it was not significant.

Results of aged vs immediately tested specimens and influence of the type of luting material can be seen in Tables 6 and 7. The results showed statistically significant differences between the aged and non-aged specimens as well as the treatments used (p < 0.05), but there was no interaction between these two factors (Table 6). Groups that were not subjected to thermal aging showed significantly higher values (Table 7).

**Table 6:** Two-way ANOVA factors for push-out test data

	df	SS	Ms	F	p-value
Cycling	1	839.73	839.729	33.94	p < **0.001**
Treatment	1	508.71	508.714	20.56	p < **0.001**
Cycling x treatment	1	2.18	2.175	0.09	0.770
Error	20	494.85	24.742		
Total	23	1845.47			

p-value in bold indicates significant difference in SBS (p < 0.05). df: degrees of freedom; SS: sum of squares; Ms: mean square.

**Table 7:** Tukey’s test for push-out bond-strength data (in MPa) of samples submitted or not to thermal aging

Thermocycling	Mean	N		
Without	63.4550 (8.59)	12	A	
With	51.6247 (6.90)	12		B

Different letters indicate statistically significant differences.

## DISCUSSION

This study aimed to evaluate the polymerization effectiveness of a device made of optical fibers, as well as the creation of a new intraradicular post that would enable photoactivation down to the apical root region. The first hypothesis was rejected, since the groups in which the optical fiber device were used showed superior bond strength in the apical portion when compared to the other groups, regardless of the resin cement used. The second hypothesis was accepted, since bond strengths were lower for all groups after thermocycling. The third hypothesis was rejected, as the light-cured luting material yielded higher bond strengths than did the dual-cured luting material.

Attenuation of light in the apical region of the root canal is a major concern among clinicians when cementing a fiberglass post, especially regarding the bond longevity. Therefore, several studies have sought strategies to increase the bond between post/luting material/dentin, such as the study by Spicciarelli et al,^23^ which evaluated two techniques that involved adhesives during the luting of the intraradicular post. According to those authors, universal adhesives represent a good alternative to conventional etch-and-rinse adhesives for fiber post luting. However, there is a lack of data regarding improved post designs in terms of bond strength.

In this study design, for the manufacture of fiberglass posts, a virtual evaluation of different prototypes was carried out through 3D finite element analysis to determine the mechanical response of hollow experimental posts and conventional posts. Taking into account that the stress magnitude around the post would not be significantly increased by the central space, it was possible to manufacture specimens based on the dimensions reported before. In addition to take stress generation into account when making the intraradicular post, it is necessary to consider the post’s partial translucency to facilitate light transmission.^17^ According to a previous report,^17^ a more translucent post would increase light passage in comparison to less translucent posts. Therefore, the post designed in the present study was similar to the post described by Giudice et al,^9^ who evaluated the mechanical strength of hollow fiberglass posts compared to hollow fiberglass posts filled with dual-curing luting material, and concluded that both groups could be viable options to restore endodontically treated teeth. The present study complements the study by Giudice et al,^9^ suggesting that a hollow up to 1.0 mm in diameter could be used, as well as light curing with the aid of an optical-fiber device to improve the bond strength of the post.

Therefore, the objective of the present study was to increase the light transmission along the entire post length through the optical fiber, as shown by Stylianou et al,^26^ who compared experimental posts containing optical fibers with conventional posts. Their results showed that polymerization through the experimental fiber was more efficient, as it provided greater light transmission and radiopacity. The present study corroborates these results,^26^ showing that the groups with better light transmission (with the aid of optical fiber) had improved bond strengths.

Dual-curing luting materials are considered the most suitable material use in for intraradicular post, as they do not depend on the direct reach of light and allow the polymerization of the resin luting material in deeper regions; however, the bond strength can be hampered by the acid-base components.^3^ In contrast, a study by Lima et al^13^ showed that the use of a dual-curing system increased the bond strength between the restorative material and dentin, even when a chemically activated luting material was used. Therefore, photoactivated luting material was used in the present study to increase its bond strength, making it applicable in luting fiberglass posts. From these in-vitro findings, it was possible to observe that the optical-fiber device and the experimental post had better results compared to the groups that were directly photoactivated.

In this study, statistically significant differences were found between the types of luting material, as well as the use (or not) of the optical fiber at different depths of the root canal. The interaction between luting material and depth was also significant, with the dual-curing luting material groups showing the lowest bond strengths, regardless of the use of the optical-fiber device. The stereomicroscopic analysis showed that adhesive failures were predominant in the groups in which the optical fiber was used, regardless of the luting material. However, for the groups that underwent thermocycling, the optical-fiber device positively influenced the bond between post/luting material/dentin analogue.

When analyzing the DC, the GD and GF groups had the lowest and highest degrees of conversion, respectively. The lower the DC, the lower the hardness of the resin luting material, and consequently the lower the bond strength between the post/luting material/dentin. According to a previous study,^21^ the greater the distance from the activating tip and the lower the post translucency, the lower the DC and consequently the bond strength, which is in agreement with the results of this study.

There was a decrease in bond strength for all groups submitted to thermal cycling, which is in agreement with the literature.^13,24^ Therefore, the present study suggests that the hollow post design is also beneficial for long-term bond strength, when light curing is performed with the aid of an optical-fiber device. In addition, further studies should be conducted to evaluate the effect of adhesive layer curing using an optical-fiber device.

According to a literature review of bond strength testing, if small-sized specimens are used in the push-out bond strength test, uniform stress distribution is favored, local differences in bonding conditions can be discerned, and the number of specimen needed for the test can be reduced.^10^ As the present investigation used 2-mm-thick specimens, more specimens were need for statistical purposes. Therefore, it is expected that the use of slices thinner than 2 mm would not present different results from those of this study in terms of mechanical behavior, but this hypothesis still needs to be tested.

As this is preliminary research, further studies aiming to improve hollow post prototypes should be performed, evaluating their fatigue performance during chewing loads, mechanical behavior in weakened roots, as well as the association with different root morphologies and inclinations. The proposal for a technical change in the manufacturing of prefabricated posts should be based on compiled data from the literature, and not only on the present study. In addition, new protocols should be developed to increase the feasibility of the proposed hollow-post design.

## CONCLUSION

The hollow fiberglass post is a promising option when cemented with light-curing luting material associated with the optical-fiber accessory tip, as it showed superior bond strength compared to those cemented with dual-curing luting material. There was a significant reduction in bond strength after aging, regardless of the luting protocol.

## Clinical relevance:

This study designed and evaluated a new fiberglass post that can overcome limitations regarding long-term bond strength and polymerization in the most apical portion of the posts.
